# Sleep Quality and Levodopa Intestinal Gel Infusion in Parkinson's Disease: A Pilot Study

**DOI:** 10.1155/2018/8691495

**Published:** 2018-11-01

**Authors:** Oriol De Fabregues, Alex Ferré, Odile Romero, Manuel Quintana, José Álvarez-Sabin

**Affiliations:** ^1^Movement Disorders Unit, Department of Neurology, Hospital Universitari Vall D'Hebron, Neurodegenerative Diseases Research Group-Vall D'Hebron Research Institute, Universitat Autònoma de Barcelona, Barcelona, Spain; ^2^Sleep Unit, Department of Neurophysiology, Hospital Universitari Vall D'Hebron, Barcelona, Spain

## Abstract

**Background:**

Sleep problems in patients with advanced Parkinson's disease (PD) have a deleterious impact on quality of life.

**Objective:**

To assess the effect of levodopa-carbidopa intestinal gel (LCIG) infusion on sleep quality in advanced PD patients.

**Methods:**

Seven patients participated in a prospective pilot study. Before and after 6 months of LCIG infusion, an overnight polysomnography was performed and the Epworth Sleepiness Scale, fatigue scale, Pittsburgh Sleep Quality Index, Beck Depression Inventory, and the Hamilton Anxiety Rating Scale were administered.

**Results:**

PSG showed low sleep efficiency. REM sleep without atony was found in 5 patients. After 6 months of LCIG infusion, the percentage of REM sleep decreased as well as the number of arousals especially due to reduction of spontaneous arousals and periodic leg movements during REM sleep, but differences were not statistically significant. Also, scores of all study questionnaires showed a tendency to improve.

**Conclusion:**

The results show a trend toward an improvement of sleep quality after 6 months of LCIG infusion, although differences as compared to pretreatment values were not statistically significant. The sleep architecture was not modified by LCIG. Further studies with larger study samples are needed to confirm these preliminary findings.

## 1. Introduction

Sleep problems are important nonmotor manifestations in patients with Parkinson's disease (PD) at different stages during the course of the disease. Numerous forms of alterations of physiologic sleep patterns have been reported, ranging from increased daytime sleepiness after introduction of a dopamine agonist to the therapeutic regimen to specific sleep-related diagnoses (e.g., restless legs syndrome, rapid eye movement sleep behavior disorder, and periodic limb movements in sleep) or sleep-related breathing disorders (e.g., obstructive sleep apnea) [1–3]. The origin of these sleep disorders is multifactorial including degeneration of the brain areas that modulate sleep, the symptoms of the disease, and the effect of medications [2].

These disturbances can primarily affect the patient's quality of life and may worsen the symptoms of PD [4, 5]. In studies that examine the impact of PD on quality of life, sleep difficulties are independent an important predictor of poor quality of life [6]. In fact, sleep disturbance, depression, and lack of independence are the primary determinants of poor quality of life [5]. Also, sleep disturbances contribute to excessive daytime sleepiness and poor daytime functioning as well as patients' reduced enthusiasm for daily events. Adverse effects have also been observed in the sleep habits and the quality of life of their caregivers [7].

There is little evidence of the impact of treatment modalities for advanced PD on sleep [8]. In relation to deep brain stimulation (DBS), a positive effect of subthalamic DBS on sleep-wake disturbances was found in a systematic review of 38 studies involving 1443 subjects [9]. However, only seven studies used polysomnography (PSG) to objectively assess sleep parameters. In a small pilot study, pallidal DBS showed improving trends in several PSG measures including sleep efficiency and latency to sleep onset [10]. Continuous levodopa-carbidopa intestinal gel (LCIG) infusion is a therapeutic option for advanced PD patients complicated with motor fluctuations refractory to conventional treatment. It has been shown that LCIG infusion improves nonmotor symptoms and quality of life [11]. The specific effects of LCIG infusion on sleep disturbances have been poorly studied. In a small clinical series of 12 PD patients, subjective measures of sleep quality and daytime sleepiness improved with LCIG infusion, although PSG was not performed [12]. There is only one open-label pilot study with a sample size limited to 11 patients that examined PSG characteristics in PD patients on a stable LCIG dose [13]. Main findings included improvement of subjective sleep quality, motor complications, and activities of daily living. PSG showed a reduction of the number of awakenings in sleep, a trend towards a lower apnea-hypopnea index, and no change in sleep latency, total sleep time and sleep efficiency [13].

The present clinical study was conducted to add evidence of sleep disturbances in advanced PD patients treated with LCIG infusion. The objective was to determine whether treatment with LCIG infusion had a beneficial effect on the quality of sleep in patients with advanced PD. In these patients, an overnight PSG was performed before and after 6 months of LCIG infusion therapy. The quality of sleep is a complex phenomenon, the assessment of which should integrate subjective and quantitative objective measures. For this reason, besides overnight PSG, we also evaluated subjective parameters using a series of validated questionnaires.

## 2. Patients and Methods

### 2.1. Study Population

Seven consecutive patients with advanced PD were included in a single-center, open-label prospectively pilot study, and were evaluated at baseline and after 6 months of LCIG infusion. The study was conducted in compliance with the ethical standards and was approved by the Ethics Committee for Clinical Research of Hospital Universitari Vall d'Hebron, Barcelona (Spain) and followed the Spanish Law 15/1999 on Personal Character Data Protection concerning confidentiality of Patient's data. The Institutional Review Board Clinical Study registration number was PR(AG)129/2008, and the Clinical Study registration number is NCT03602924. Written informed consent was obtained from all patients.

Patients started LCIG infusion after receiving an implant of percutaneous endoscopic gastrostomy with jejunal extension (PEG-J) following a previously described procedure used in our center [14]. The initial LCIG maintenance dose was calculated according to the levodopa equivalent daily dose; the optimal dose was titrated individually until reaching the best motor performance, controlling motor fluctuations without causing annoying dyskinesia, and getting a stable infusion for less than 16 hours a day, stopped at night, when patients received an oral nocturnal dose of levodopa.

### 2.2. Study Procedures

All participants underwent a full overnight PSG at the Sleep Unit of our institution. The recorded parameters included electroencephalography (EEG), electro-occulography (EOG), electromyography (EMG), electrocardiography (ECG), respiratory effort, oronasal airflow, oxygen saturation, snoring sounds, and body position. Sleep scoring was performed by a trained technician according to the American Academy of Sleep Medicine (AASM) scoring criteria 2012 [15]. The reported parameters included sleep efficiency, wake after sleep onset (WASO), sleep latency, REM latency, rapid-eye movement (REM) sleep, nonREM (NREM) sleep (stages 1–3), snoring sounds, apnea-hypopnea index (AHI), arousal index, spontaneous arousals, respiratory effort-related arousals (RERA), leg movement arousals, periodic leg movements in sleep (PLMS), PLMS in REM and NREM, oxygen saturation (SpO_2_), and CT90.

### 2.3. Assessments

Evaluation included the Epworth Sleepiness Scale (ESS) [16], the Fatigue Severity Scale (FSS) [17], the Pittsburgh Sleep Quality Index (PSQI) [18], sleep efficiency, the Beck Depression Inventory (BDI) [19], and the Hamilton Anxiety Rating Scale (HARS) [20]. Also, complete pharmacological data, including antiparkinsonian drugs and treatments potentially influencing sleep and daytime sleepiness (i.e., clonazepam, quetiapine, and serotonin selective reuptake inhibitors) were collected at baseline and at follow-up.

The ESS measures the general level of daytime sleepiness (the sum of 8 item scores, 0–3) with a total score ranging from 0 to 24. The higher the ESS score, the higher the person's average sleep propensity in daily life. The FSS is a 9-item questionnaire with questions related to how fatigue interferes with certain activities and rates its severity. Items are scored on a 7 point scale, with 1 = strongly disagree and 7 = strongly agree, with a total score ranging between 9 and 63. The higher the score, the greater fatigue severity. The PSQI consists of 19 individual items creating 7 component scores and one composite score. Each item is weighted on a 0–3 interval scale. The global PSQI score is then calculated by totaling the 7 component scores, providing an overall score ranging from 0 to 21, where lower scores denote a healthier sleep quality. Subjective sleep efficiency (component #4 of the PSQI) was calculated as number of hours slept/number of hours spent in bed × 100 and expressed as a percentage. The BDI consists of 21 questions about how the subject has been feeling during the last week, and a value of 0 to 3 is assigned for each answer, with total score ranging from 0 to 63 (0–13 minimal, 14–19 mild, 20–28 moderate, 29–63 severe). The HARS consists of 14 items, each of which contains a number of symptoms, which are rated scale of 0 to 4, with 4 being the most severe. The total score ranges from 0 to 56.

Moreover, motor fluctuations, dyskinesia, and other motor and nonmotor clinical aspects were evaluated before starting LCIG infusion (baseline) after 6 months of treatment. Motor fluctuations were assessed by “off” time, in hours, recorded in Parkinson's Disease Diary©. Dyskinesia and other motor symptoms were evaluated using the Unified Parkinson's Disease Rating Scale (UPDRS) [21] part IV–Complications of Therapy, UPDRS part II–Activities of Daily Living in On and Off, UPDRS part III–Motor Examination in On and Off; Hoehn and Yahr stage in On and Off [22]; and the Schwab and England Activities of Daily Living (ADL) scale in On [23]. Nonmotor clinical aspects evaluated were cognitive function using the Mini Mental State Examination (MMSE) test [24] and UPDRS part I–Mental, Behavior, and Mood.

Complete pharmacological data, including antiparkinsonian drugs and treatments potentially influencing sleep and daytime sleepiness (i.e., benzodiazepines (clonazepam, lorazepam), neuroleptic (quetiapine), and serotonin selective reuptake inhibitors) were, recorded at baseline and follow-up.

### 2.4. Statistical Analysis

Data are expressed as mean and standard deviation (±SD). The Wilcoxon signed-rank test was used for the comparison of paired samples before and after 6 months of LCIG infusion. Statistical analysis was performed with the SPSS version 17.0 (Statistical Package for Social Sciences, SPSS, Inc., Chicago, IL, USA). Statistical significance was set at *P* < 0.05.

## 3. Results

We studied 6 women and 1 man diagnosed with advanced PD, with a mean age of 69.6 years (range 60–78) and a mean body mass index (BMI) of 24.5 kg/m^2^ (range 20–32).

Results of clinical variables are shown in Table 1. A significant improvement in motor fluctuations was observed (daily mean “off” time decreased from 6.3 ± 1.4 h at baseline to 1.1 ± 0.7 h after 6 months, *P* < 0.001). Motor symptoms evaluated with UPDRS part III remained stable and the mean score in “off” stage did not change in any of the patients. Dyskinesia assessed with UPDRS part IV decreased significantly from 5.4 ± 2.4 at baseline to 2.9 ± 1.1 after 6 months of LCIG treatment (*P*=0.028). None of the patients presented a worsening in the percentage of the waking time with dyskinesia, which was reduced in 2 patients (28.6%) and remained stable in the remaining 5 patients (71.4%). The severity of dyskinesias improved in 3 (42.9%) patients, remained stable in 3 (42.9%), and worsened in 1 (14.3%). Nonmotor cognitive function (UPDRS part I) tended to improve.

As shown in Table 2, at 6 months after LCIG infusion, there was a decrease in scores of the ESS, FSS, and PSQI as compared with baseline, indicating an improvement in daytime sleepiness, fatigue, and sleep quality. Severe score of the PSQI at baseline was recorded in 4 patients (57.1%), 3 of which changed to a moderate score at follow-up. One patient with moderate score at baseline had a normal score at follow-up, and of 2 patients with normal score at baseline, 1 had a mild score at follow-up. Sleep efficiency also improved. Differences, however, were not statistically significant. Changes in the scores of BDI and HARS were not significant either.

Results of PSG showed low generalized sleep efficiency. No significant differences were observed in sleep macrostructure parameters, respiratory events, or periodic leg movements. There was a decrease in the percentage of REM sleep (16.2 ± 9.9% vs. 10.4 ± 6.8%, *P*=0.080) and arousal index (15.0 ± 7.0 vs. 12.9 ± 5.6, *P*=0.115) especially due to reduction of spontaneous arousals (7.5 ± 3.1 vs. 5.2 ± 5.0, *P*=0.075) and PLMS during REM sleep (20.7 ± 31.6 vs. 2.9 ± 3.7, *P*=0.285), but differences were not statistically significant. The percentage of NREM sleep increased from a mean of 83.6 ± 10.1% at baseline to 89.6 ± 6.8% after 6 months of LCIG infusion therapy, although differences did not reach statistical significance (*P*=0.080) (Table 3).

The patients had no previous treatment at the beginning of the infusion with dopamine agonists. Also, monoamine oxidase (Mao) and catechol-O-methyl transferase (COMT) inhibitors were withdrawn in all patients, whereas the use of other drugs potentially influencing the quality of sleep and daytime sleepiness were allowed. The number of patients taken drugs with hypnotic effect before and after treatment with LCIG infusion remained unchanged (Table 1).

## 4. Discussion

This prospective pilot study in a reduced number of patients with advanced PD treated with LCIG infusion shows that their quality of sleep is poor. Treatment with LCIG infusion did not aggravate the quality of sleep in these patients. We found a decreased time in REM sleep and a tendency of improvement in the number of arousals especially in relation to a reduction of spontaneous arousals and PLMS during REM sleep. Despite a reduction in the percentage of REM sleep, the quality of sleep seems to be better as shown by a decrease of spontaneous arousals and PLMS. These changes in objective parameters may explain the trend towards improvement of subjective measures. Although statistically significant differences in clinical or polysomnographic parameters were not found due to the limited population of 7 patients with advanced PD, the present results regarding maintenance of sleep architecture and a trend toward an improvement of sleep quality after 6 months of LCIG infusion, are similar to results reported by Zibetti et al. [13]. In this respect, two pilot studies with a small sample size (11 patients in the study of Zibetti et al. [13] and 7 in our study) point toward similar findings of amelioration of sleep parameters in advanced PD patients treated with LCIG infusion. In our study, however, differences of objective and subjective variables were not statistically significant. The most likely explanation is that the number of subjects (7 patients) is insufficient to reach statistical significance in any of the PSG measures presented.

Various studies have demonstrated an improvement in nonmotor symptoms, including the sleep/fatigue domain of the Non-Motor Symptoms Scale (NMSS) and quality of life in advanced PD patients treated with LCIG infusion [11, 25–28]. However, based on a recent systematic review of randomized controlled trials (RCTs) and observational studies collected from PubMed and EMBASE until March 2016, the quality of evidence regarding effectiveness of LCIG infusion in improving quality of life is moderate and for reducing nonmotor symptoms is low [29]. Also, there is no evidence of the effectiveness of LCIG infusion in the treatment of subjective fatigue [30].

There is little information on the specific effect of LCIG infusion on the quality of sleep in patients with advanced PD. In a study by Zibetti et al. [12] carried out in a sample of 12 patients, sleep and nocturnal symptoms were evaluated with the modified version of Parkinson's Disease Sleep Scale (PDSS-2) Daytime sleepiness was also assessed with the ESS. A significant improvement in all study variables (PDSS-2 total score, disturbed sleep, motor symptoms at night, PD symptoms at night, and ESS score) was found at 2–4 months of follow-up after starting LCIG infusion therapy. Honig et al. [11] evaluated 24 patients with advanced PD who switched from oral medications to LCIG and were followed for 6 months. Treatment with LCIG reduced motor fluctuations and dyskinesias with statistically significant decreases of the nonmotor symptoms scale (NMSS) and improvement in quality of life (PDQ-8 questionnaire). These results are consistent with the present findings. Although our data should be interpreted considering the reduced number of patients included in the study, it was shown that treatment with LCIG infusion was not associated with a significant amelioration of sleep quality. Overall, the quality of sleep in our patients was poor, but it was not found to be worsened by LCIG infusion therapy.

## 5. Conclusions

The main finding of this preliminary study of 7 advanced PD patients treated with LCGI is a trend toward an improvement of sleep quality after 6 months of LCIG infusion, although differences as compared to pretreatment values were not statistically significant. The sleep architecture was not modified by LCIG. Further prospective masked studies with larger series of patients on LCIG infusion therapy, not stopped at night, are necessary to clarify the positive influence of LCIG on sleep quality in patients with advanced PD.

## Figures and Tables

**Table 1:** Changes in “off” time hours, UPDRS values, cognitive function, body mass index, and pharmacological therapy at baseline and after 6 months of LCIG infusion.

Variables	Baseline	Follow-up	*P* value
Off time hours recorded in Parkinson's disease diary© (daily mean “off” time)	6.3 ± 1.4	1.1 ± 0.7	<0.001
UPDRS part IV (dyskinesia)	5.4 ± 2.4	2.9 ± 1.1	0.028
UPDRS part II (activities of daily living)			
On	11.4 ± 5.9	10.9 ± 5.6	0.742
Off	22.9 ± 8.3	22.3 ± 7.5	0.231
UPDRS part III (motor examination)			
On	18 ± 3.7	16.6 ± 4.9	0.245
Off	34.1 ± 11.1	34.1 ± 11.1	—
UPDRS part I (mentation, behavior, and mood)	3.6 ± 3.7	2.4 ± 2.1	0.156
MMSE (cognitive function)	29 (27–30)	29 (27–30)	0.317
Body mass index (BMI)	24.5 ± 4.0	23.8 ± 3.3	0.424
Bedtime drugs, no. patients			
Benzodiazepine drugs	6/7	6/7	1.000
Serotonin selective reuptake inhibitor	4/7	4/7	1.000
Neuroleptic drugs	1/7	2/7	1.000

Data as mean ± standard deviation unless otherwise stated.

**Table 2:** Changes of subjective parameters before and after 6 months of LCIG infusion therapy in 7 patients with advanced PD.

Questionnaires	LCIG infusion therapy		*P* value
	Before	At 6 months	
ESS	6.4 ± 3.6	4.7 ± 4.1	0.340
PSQI	11.9 ± 6.4	8.9 ± 5.0	0.137
Subjective sleep efficiency	61.9 ± 21.5	57.8 ± 16.0	0.612
BDI	11.7 ± 8.7	14.3 ± 9.4	0.497
HARS	21.6 ± 10.8	22.1 ± 12.4	0.917

Data as mean ± standard deviation. ESS: Epworth Sleepiness Scale; PSQI: Pittsburgh Sleep Quality Index; BDI: Beck Depression Inventory; HARS: Hamilton Anxiety Rating Scale.

**Table 3:** Comparison of polysomnographic results before and after 6 months of LCIG infusion therapy in 7 patients with advanced PD.

Variables	LCIG infusion therapy		*P* value
	Before	At 6 months	
Sleep efficiency (%)	66.7 ± 8.4	57.8 ± 16.0	0.173
Wake after sleep onset (WASO) (min)	119.9 ± 74.2	113.0 ± 54.1	0.345
Sleep latency (min)	29.7 ± 40.5	71.2 ± 97.2	0.345
REM latency (min)	147.3 ± 72.4	139.8 ± 63.1	0.893
REM sleep (%)	16.2 ± 9.9	10.4 ± 6.8	0.080
NREM sleep (%)	83.6 ± 10.2	89.6 ± 6.8	0.080
Stage 1	16.8 ± 10.0	23.8 ± 13.7	0.463
Stage 2	52.9 ± 8.4	52.4 ± 12.0	0.345
Stage 3	13.9 ± 7.5	13.0 ± 11.9	0.752
Snoring sounds (number/hour)	323.2 ± 279.3	228.6 ± 226.0	0.715
Apnea-hypopnea index (AHI)	11.8 ± 18.0	12.7 ± 14.0	0.686
Arousal index	15.0 ± 7.0	12.9 ± 5.6	0.115
Spontaneous arousals	7.5 ± 3.1	5.2 ± 5.0	0.075
Respiratory effort-related arousals (RERA)	4.9 ± 7.2	4.8 ± 4.1	0.893
Leg movement arousals	3.1 ± 2.3	2.9 ± 2.5	0.462
Periodic leg movement in sleep (PLMS)	12.5 ± 11.6	7.7 ± 11.1	0.345
PLMS in REM sleep	20.7 ± 31.6	2.9 ± 3.7	0.285
PLMS in NREM sleep	10.3 ± 9.5	8.3 ± 12.4	0.893
Oxygen saturation (SpO_2_) (%)			
Baseline	95.1 ± 2.3	95.8 ± 2.1	0.339
Mean	93.8 ± 2.0	94.3 ± 2.2	0.461
Minimum	87.0 ± 8.9	86.4 ± 6.6	0.672
Oxygen saturation <90%, CT90 (%)	2.7 ± 4.2	1.7 ± 2.0	0.416

Data as mean ± standard deviation. 6–10: higher normal daytime sleepiness. 11–12: mild excessive daytime sleepiness. 13–15: moderate excessive daytime sleepiness. 16–24: severe excessive daytime sleepiness.

## Data Availability

The data used to support the findings of this study are available from the corresponding author upon request.
